# Performance of Emergency Department Screening Criteria for an Early ECG to Identify ST‐Segment Elevation Myocardial Infarction

**DOI:** 10.1161/JAHA.116.003528

**Published:** 2017-02-23

**Authors:** Maame Yaa A. B. Yiadom, Christopher W. Baugh, Conor M. McWade, Xulei Liu, Kyoung Jun Song, Brian W. Patterson, Cathy A. Jenkins, Mary Tanski, Angela M. Mills, Gilberto Salazar, Thomas J. Wang, Robert S. Dittus, Dandan Liu, Alan B. Storrow

**Affiliations:** ^1^ Vanderbilt University Nashville TN; ^2^ Department of Emergency Medicine Brigham and Women's Hospital Boston MA; ^3^ Department of Emergency Medicine University of California at Davis Sacramento CA; ^4^ Department of Emergency Medicine University of Wisconsin at Madison WI; ^5^ Department of Emergency Medicine Oregon Health & Sciences University Portland OR; ^6^ Department of Emergency Medicine University of Pennsylvania Philadelphia PA; ^7^ Department of Emergency Medicine University of Texas Southwestern Dallas TX

**Keywords:** emergency department, false‐negative rate, informedness, missed case rate, screening, ST‐segment elevation myocardial infarction, timely care, Clinical Studies, Ischemia, Vascular Disease, Electrocardiology (ECG), Diagnostic Testing

## Abstract

**Background:**

Timely diagnosis of ST‐segment elevation myocardial infarction (STEMI) in the emergency department (ED) is made solely by ECG. Obtaining this test within 10 minutes of ED arrival is critical to achieving the best outcomes. We investigated variability in the timely identification of STEMI across institutions and whether performance variation was associated with the ED characteristics, the comprehensiveness of screening criteria, and the STEMI screening processes.

**Methods and Results:**

We examined STEMI screening performance in 7 EDs, with the missed case rate (MCR) as our primary end point. The MCR is the proportion of primarily screened ED patients diagnosed with STEMI who did not receive an ECG within 15 minutes of ED arrival. STEMI was defined by hospital discharge diagnosis. Relationships between the MCR and ED characteristics, screening criteria, and STEMI screening processes were assessed, along with differences in door‐to‐ECG times for captured versus missed patients. The overall MCR for all 7 EDs was 12.8%. The lowest and highest MCRs were 3.4% and 32.6%, respectively. The mean difference in door‐to‐ECG times for captured and missed patients was 31 minutes, with a range of 14 to 80 minutes of additional myocardial ischemia time for missed cases. The prevalence of primarily screened ED STEMIs was 0.09%. EDs with the greatest *informedness* (sensitivity+specificity−1) demonstrated superior performance across all other screening measures.

**Conclusions:**

The 29.2% difference in MCRs between the highest and lowest performing EDs demonstrates room for improving timely STEMI identification among primarily screened ED patients. The MCR and informedness can be used to compare screening across EDs and to understand variable performance.

## Introduction

The diagnosis of ST‐segment elevation myocardial infarction (STEMI) in the emergency department (ED) is made solely by ECG. Timely diagnosis is critical to achieving timely intervention. The goal is to achieve a door‐to‐ECG time of 10 minutes.^1^ The first 10 minutes of an ED visit, however, are administrative. To achieve timely diagnosis of patients with potential STEMI, EDs use early ECG screening criteria for all ED patients on arrival and prior to the physician evaluation. Different EDs, however, have different criteria. The association of these criteria with missed STEMI cases and the impact of missed screening on myocardial ischemia time are unknown.

**Figure 1:**
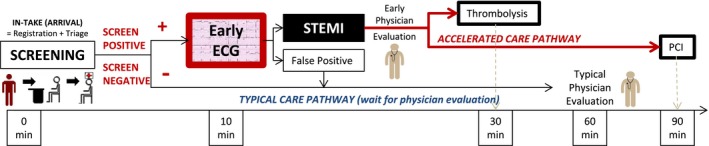
Timely care goals for STEMI screening, diagnosis, and treatment: emergency department arrival to treatment. PCI indicates percutaneous coronary intervention; STEMI, ST‐segment elevation myocardial infarction.

**Figure 2:**
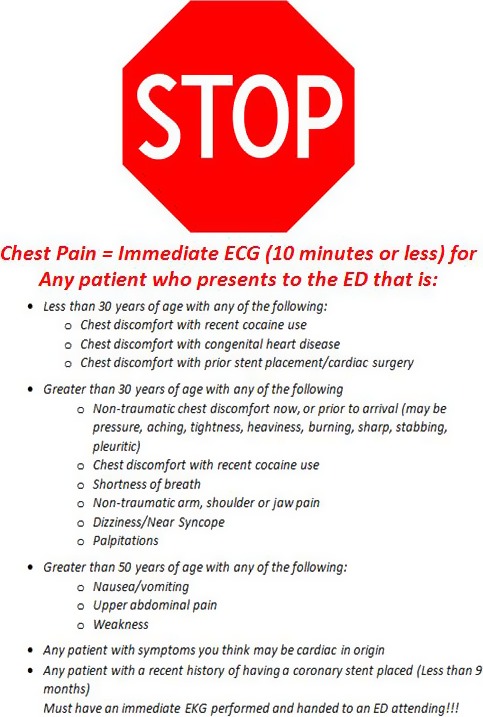
Sample emergency department early ECG screening criteria to screen for ST‐segment elevation myocardial infarction. ED indicates emergency department.

The first 10 minutes of an ED visit typically consist of intake processes (registration and triage) that usually occur well before a physician encounter (Figure 1). Consequently, ED registration and triage staff use preestablished screening criteria to identify patients that should receive an early ECG to diagnose STEMI (Figure 2). Given the time‐sensitive nature of STEMI care, failure to identify candidates for an early ECG during ED intake processes subjects patients to diagnostic and potential treatment delay.^1^, ^2^, ^3^ Patients eventually diagnosed with STEMI present with a wide spectrum of symptoms. STEMI screening criteria are preestablished algorithms using a patient's arrival information. The criterion within the ED screening criteria falls on the spectrum of including only “chest pain” as the most typical symptom and variably includes consideration for more atypical symptoms or age. Identifying the most sensitive approach, balanced with specificity, could guide EDs in optimizing their STEMI screening performance.

### Background

Despite decades of quality‐improvement efforts, the timely diagnosis of STEMI patients in the ED presents significant operational challenges. In managing the timely care of patients with STEMI, EDs encounter 3 distinct types of patients who differ on the screening, diagnosis, and treatment continuum. First are patients who have been prediagnosed with STEMI, who have had an ECG performed and interpreted by another provider. They are usually transferred to an ED or a hospital capable of definitive treatment, most often percutaneous coronary intervention. The expectation is that treatment will be initiated on arrival. Second are prescreened patients who have been evaluated by another provider and referred to the ED with an ECG concerning for ischemia. In these cases, the diagnosis needs to be confirmed with a repeat ECG or reinterpretation of the ECG on arrival. The burden of screening for STEMI from the report of symptoms has already been met in these 2 scenarios; however, treatment has not been initiated. Third are the patients at greatest risk of delays in care: those arriving in the ED with undifferentiated symptoms who are primarily screened by that ED. In these cases, timely STEMI diagnosis is dependent on the patient's condition and symptoms triggering ED intake staff to perform an early ECG.^4^, ^5^ The ECG is often considered the screening test for STEMI. In the context of emergency care, however, treatment is initiated with ECG evidence of STEMI, making it a diagnostic test.^6^ The true screening test is the criteria by which ED care providers initiate an ECG (Figure 2).

Overly comprehensive screening criteria for a rare disease like STEMI risks underutilization. The sole use of chest pain is user friendly but likely produces inadequate case capture. Overall, 20% to 30% of patients with STEMI will report atypical symptoms like shortness of breath and dizziness^7^, ^8^, ^9^, ^10^, ^11^ or will focus on associated symptoms like jaw, neck, or back pain. In addition, age positively influences the probability of STEMI, and elderly patients with STEMI more frequently report atypical symptoms.^12^, ^13^, ^14^ Shorter time to reperfusion has been shown to improve outcomes.^15^, ^16^, ^17^, ^18^, ^19^ Door‐to‐ECG time interval is the first STEMI care target toward achieving timely intervention.

### Study Objective

We examined patient‐oriented outcomes associated with screening performance variation for primarily ED‐screened STEMI patients and the impact of a false‐negative screen on myocardial ischemia time. Little is known about performance variation in contemporary ED STEMI screening, the influence of the processes that occur between ED arrival and diagnosis, or the effect of more comprehensive criteria on the quality of screening. This evidence gap was the focus of our investigation.

## Methods

### Study Design and Setting

**Figure 3:**
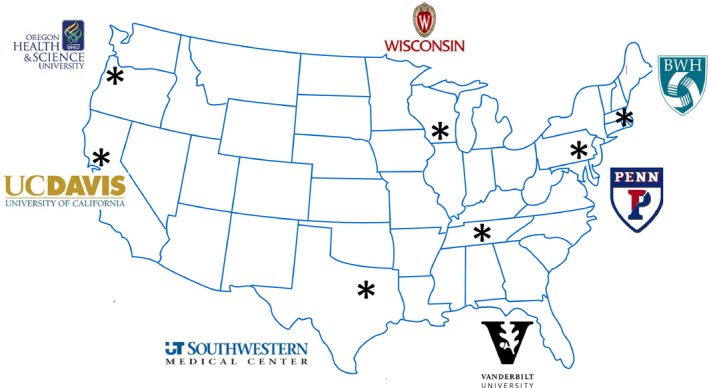
Study site geographic and population density distribution.

This prospective historical cohort study compared the 2014 STEMI screening performance of 7 EDs (University of Pennsylvania, Brigham and Women's Hospital, Vanderbilt University, University of Wisconsin, Parkland Hospital at the University of Texas Southwestern, Oregon Health and Sciences University, and University of California Davis) selected for their geographic diversity, with considerations for US population density (see Figure 3). Our geographic and population distribution sample of US EDs and patients was selected to reduce spectrum bias from regional practice variation. The institutional review boards of all 7 participating institutions granted ethics approval for this investigation, with Vanderbilt University serving as the data coordinating center. Patient consent was waived at each institution, given the use of deidentified patient data to measure ED‐level screening performance (the full study protocol is available in Data S1 and Tables S1 and S2).

### Data Source and Data Collection

Data sources included summative case counts obtained from ED and hospital electronic medical record (EMR) system data reports from each hospital. Prior to data extraction, each site primary investigator met with the hospital's EMR information technology liaison to ensure that data could be obtained according to the study's data dictionary, protocol, inclusion criteria, and exclusion criteria. This involved the EMR information technology liaison developing programming code to create a data report to reliably extract the required case counts from the EMR system. The study was launched only after we received confirmation that this consistency could be achieved at all sites. To verify accuracy, each site obtained summative case counts from the hospital STEMI case review committees. Any discrepancies were adjudicated by each site primary investigator via chart review. Each institution reported data using an online data collection tool developed within REDCap, a secure, widely used, Web‐based data collection and database management application.^20^ For the study data collection tool, see Data S1 (https://redcap.vanderbilt.edu/surveys/?s=ET3WK99HDM).

### STEMI Screening Criteria, Process, and Diagnosis

The content of each ED's screening criteria was classified into 5 categories: chest pain, atypical symptoms, associated symptoms, consideration of an age threshold, and other. Criteria comprehensiveness was defined as the number of criteria included by each ED. We qualitatively assessed the STEMI screening process by identifying the first points of contact (electronic check‐in kiosk, clerks, nurses, or midlevel providers or physicians) performing early ECG screening for patients arriving via emergency medical services (EMS) versus self‐transport. We confirmed that all 7 sites had established screening criteria (documented by policy or protocols for patient intake) as a fixed component of the ED patient intake and triage process and that neither the criteria nor the processes changed during the study period.

Using historical EMR data, we defined a positive screening result as having an early ECG within 15 minutes of arrival. ED arrival was defined as the first documented presence or time stamp of the patient's presence in the ED. The total number of ED patients seen included all persons registered as ED patients, including those who ultimately eloped, left without being seen, left before completing treatment, or left against medical advice. ED STEMI cases included all patients with an ED or hospital discharge diagnosis of STEMI.^21^, ^22^ Our study door‐to‐ECG target was more generous than guideline recommendations; however, it reduced the likelihood that potential electronic time stamp delays would overestimate the number of missed cases. Fifteen minutes also permitted us to capture screened patients at a threshold well below the shortest mean door‐to‐doctor time among the participating EDs to avoid including ECGs completed after intake. To reduce misclassification bias, STEMI was defined using *International Classification of Diseases, Ninth Revision* codes previously used in the literature (410.01, 410.11, 410.21, 410.31, 410.41, 410.51, 410.61, 410.81, 410.91).^13^, ^23^

### Patient Population Selection

We defined eligible ED visits as those for all patients aged ≥18 years who were registered and evaluated in the ED from January 1 to December 31, 2014, to eliminate enrollment bias across sites using different EMR systems with different data‐reporting functions. To reduce misclassification bias, we excluded prediagnosed and prescreened patients by excluding patients who arrived with an ECG from another facility or from a transporting EMS team that was not repeated in the receiving ED.

### Study Outcomes: Screening Performance Measures

We calculated the diagnostic test characteristics for STEMI screening for each individual ED and for the whole study population using ED‐reported summative case counts for the total number of ED patients seen, early ECGs performed within 15 minutes of arrival (ie, positive screen), undifferentiated STEMI cases evaluated in the ED (ie, cases), and undifferentiated STEMI cases that did not receive an early ECG (ie, missed screening cases). The missed case rate (MCR), or the proportion of patients with a final diagnosis of STEMI that did not receive a timely early ECG (false‐negative rate or type II error rate), was our primary performance measure outcome. We included sensitivity, specificity, accuracy, diagnostic odds ratio, and *informedness* as additional measures.^22^ Informedness is a measure that is analogous to Youden's point on a receiver operating characteristic curve. It captures the balance between a test's sensitivity and specificity (sensitivity+specificity−1) and summarizes the performance of a diagnostic test (values range from −1 to 1 with 0 representing a useless test and 1 indicating a perfect test with no false positives or false negatives).^24^ We included positive and negative predictive values to be complete; however, these measures were not expected to differentiate the quality of ED screening performance, given the low frequency of STEMI.^25^

### Other ED Characteristics

The median door‐to‐ECG time and interquartile range was reported by each ED for all primarily screened STEMI patients in addition to the subgroups of captured cases (screened positive) and missed cases (screened negative). In addition, we calculated the proportion of ED patients who were evaluated for myocardial ischemia as the myocardial ischemia work‐up rate to account for the frequency with which myocardial ischemia was considered in the local ED population. This rate was a proxy for the familiarity of the staff with the signs of ischemia. It included all STEMI patients as well as those who received both an ECG and serum cardiac troponin testing during their ED stay.

The reported STEMI‐related clinical operations data included each institution's formalized early ECG screening criteria for STEMI (with provision of the ED policy for study team verification), total number of ED patients seen, early ECGs performed within 15 minutes of arrival (ie, screen positive), undifferentiated STEMI cases evaluated in the ED, and undifferentiated STEMI cases that did not receive an early ECG (ie, missed screening cases). This is in addition to the median door‐to‐ECG time and interquartile range for all primarily screened STEMI patients, the proportion of ED patients who received both an ECG and a serum cardiac troponin level during the ED visit (the myocardial ischemia evaluation rate), the first contact for ED patients who arrived via EMS and non‐EMS transportation (STEMI screening agent), and the person who recorded the chief complaint of ED patients who arrived via EMS and non‐EMS transportation. Demographic descriptors collected for each ED included annual patient volume and case mix index as measures of patient population acuity and complexity.^26^

### Statistical Analysis

With a total of 7 EDs, we performed unadjusted analyses to explore the relationship among the STEMI screening performance measures, with a focus on the MCR and factors anticipated to influence screening performance including (1) ED characteristics such as primarily screened STEMI incidence, ED volume, frequency of myocardial ischemia evaluation, and case mix index; (2) comprehensiveness of screening criteria; and (3) screening processes. We calculated Spearman rank correlation coefficients between continuous factors and the MCR as well as other STEMI screening performance measures. The Kruskal–Wallis test was used to compare STEMI screening performance across levels of categorical factors. Comprehensiveness of screening criteria was measured by the number of criteria used. The categorical data components of the screening process were documented as an ED check‐in kiosk, triage registered nurse, registration clerk, greeter, and other (midlevel provider or physician). We reported frequencies, proportions, or ratios for all patient population characteristics and screening performance measures. We used the Wilcoxon signed rank test to assess whether the mean difference between each ED's median door‐to‐ECG time for captured and missed cases was significant. Scatter plots for the MCR and selected continuous factors were also presented for illustration purposes. All statistical analyses were conducted using R 3.1.2 (2015; R Foundation for Statistical Computing, https://www.R-project.org).

## Results

In total, 472 166 adult patients were primarily screened for an early ECG to diagnose STEMI; of those, 407 were diagnosed with STEMI, for a total study population prevalence of 0.09% (95% CI 0.08–0.10%) (Table 1). Study‐site STEMI prevalence ranged from 0.03% to 0.18%. This is a subset of the larger hospital STEMI population. Consequently, the prevalence of STEMI was lower than typically reported. The difference in the median door‐to‐ECG time between captured and missed primarily screened STEMI patients was 31 minutes (95% CI 9.7–52.3; *P*=0.018) of additional myocardial ischemia time (Table 2). The screening results for each ED and for the total screened population are summarized in the following sections and in Table 1.

**Table 1:** Diagnostic Performance of the Screening Criteria for an Early ECG to Diagnose STEMI

	ED 1	ED 2	ED 3	ED 4	ED 5	ED 6	ED 7	All
ED characteristics								
Annual patient volume	128 730	44 369	68 621	64 355	59 824	59 898	33 401	470 166
Primarily screened STEMI patients	43	23	75	61	74	72	59	407
Primarily screened STEMI prevalence (%)	0.03	0.05	0.11	0.09	0.12	0.12	0.18	0.09
MI work‐up rate (%)^a^	19	17	19	17	19	23	16	19
Case mix index	1.4	2.01	2.09	1.4	1.67	3.14	1.98	1.96
Test characteristics								
**Missed case rate [false negatives]** **(95% CI)**	**32.6 [14]** **(20.1–47.5)**	**21.7 [5]** **(9.7–41.9)**	**20.0 [15]** **(12.5–30.4)**	**11.5 [7]** **(5.7–21.8)**	**6.8 [5]** **(2.9– 14.9)**	**5.6 [4]** **(2.2–13.4)**	**3.4 [2]** **(0.9– 11.5)**	**12.8 [52]** **(9.9–16.4)**
True‐negatives cases	118 616	38 774	55 454	55 945	51 939	54 331	32 272	407 331
True‐positive cases	29	18	60	54	69	68	57	355
False positives (rate %)	10 071 (8)	5572 (13)	13 092 (19)	8349 (13)	7811 (13)	5495 (11)	1070 (3)	51 460 (13)
Sensitivity (%)	67.4 (52.5–79.5)	78.3 (58.1–90.3)	80.0 (69.6–87.5)	88.5 (78.2–94.33)	93.2 (85.1–97.1)	94.4 (86.6–97.8)	96.6 (96.6–97.0)	87.2 (83.6–90.1)
Specificity (%)	92.2 (92–92)	87 (87–88)	81 (81–81)	87 (87–87)	87 (87–87)	91 (91–91)	97 (72–73)	89 (86–87)
PPV (%)	0.29 (0.20–0.41)	0.32 (0.20–0.51)	0.46 (0.35–0.59)	0.64 (049–0.84)	0.88 (0.69–1.11)	1.22 (0.97–1.55)	5.06 (3.92–6.5)	0.69 (0.50–0.62)
NPV (%)	99.99 (99.98–99.99)	99.99 (99.97–99.99)	99.97 (99.96–99.98)	99.99 (99.97–99.99)	99.99 (99.98–1.00)	99.99 (99.98–1.00)	99.99 (99.98–1.00)	99.99 (99.98–99.99)
(+) Likelihood ratio	8.6 (6.7–10.2)	6.2 (4.6–7.2)	4.2 (3.6–4.6)	6.8 (6.0–7.3)	7.1 (6.5–7.5)	10.0 (9.4–11)	30 (27–32)	7.7 (7.4–8.0)
(−) Likelihood ratio	0.38 (0.25–0.54)	0.25 (0.11–0.48)	0.25 (0.15–0.38)	0.13 (0.07–0.25)	0.08 (0.03–0.17)	0.06 (0.02–0.15)	0.04 (0.01–0.12)	0.15 (0.12–0.19)
Diagnostic odd ratio	21 (12–39)	25 (9.6–65)	17 (9.7–30)	52 (23–112)	92 (38–220)	168 (64–444)	860^b^ (231–3201)	43 (31–55)
Accuracy (%)^b^	92 (92–92)	87 (87–88)	81 (81–81)	87 (87–87)	87 (87–87)	91 (91–91)	97 (97–97)	89 (89–89)
Informedness (%)^c^	57	66	61	76	80	85	97	75
Included in screening criteria for early ECG^d^								
Chest pain	Yes	Yes	Yes	Yes	Yes	Yes	Yes	100%
Atypical MI symptoms	Yes	Yes	Yes	Yes	Yes	Yes	Yes	100%
Associated MI symptoms	No	Yes	Yes	No	Yes	Yes	Yes	71%
Age threshold	Yes	Yes	Yes	Yes	Yes	Yes	No	86%
Other	No	No	Yes	No	Yes	No	No	29%
Total criteria used	3	4	5	3	5	4	3	
ED patient contact in first 10 minutes^e^								
EMS arrival first contact	Kiosk	Other	Triage RN	Triage RN	Triage RN	Triage RN	Triage RN	
Walk‐in arrival first contact	Kiosk	Reg Clerk	Reg Clerk	Triage RN	Greeter	Reg Clerk	Triage RN	
Chief complaint recorded for EMS arrivals	Other	Other	Triage RN	Triage RN	Triage RN	Triage RN	Triage RN	
Chief complaint recorded for walk‐ins	Triage RN	Reg Clerk	Reg Clerk	Triage RN	Triage RN	Triage RN	Triage RN	
Chief complaint recorded for EMS arrivals	Other	Other	Triage RN	Triage RN	Triage RN	Triage RN	Triage RN	

ED indicates emergency department; EMS, emergency medical services; MI, myocardial infarction; NPV, negative predictive value; PPV, positive predictive value; Reg, registration; RN, registered nurse; STEMI, ST‐segment elevation myocardial infarction.

a MI work‐up rate=total acute coronary syndrome evaluations=troponin and ECG at any time during the ED visit.

b Accuracy=(true positive+true negative)/(true positive+false positive+true negative+false negative).

c Informedness (calculated as [sensitivity+specificity]−1) is analogous to the ideal cut point on a receiver operating characteristic curve in expressing a balance between the tradeoffs and the benefits of test sensitivity and specificity.

d Yes=included, no=not included.

e Options were greeter, automated check‐in kiosk, reg clerk, triage RN, midlevel provider, physician.

Numbers in parentheses represent the 95% Confidence Intervals.

**Table 2:** Difference in Door‐to‐ECG Time for Captured and Missed Cases

		All Primarily Screened ED STEMI Patients	Screen‐Positive “Trigger Positive”	Screen‐Negative “Trigger Negative”	Median Difference
	MCR, %	Median (IQR)	Median (IQR)	Median (IQR)	
ED 7	3.4	3 (1–5)	3 (1–5)	18 (17–19)	15
ED 6	5.6	9 (5–10)	7 (6–18)	33 (28–74)	26
ED 5	6.8	6 (3–9)	5 (2–8)	19 (18–26)	14
ED 4	11.5	5 (2–10)	4 (2–8)	26 (18–72)	22
ED 3	20.0	7 (4–16)	6 (4–7)	44 (19–117)	38
ED 2	21.7	5 (3–12)	5 (3–7)	27 (26–29)	22
ED 1	32.1	8 (5–17)	7 (5–8)	87 (21–149)	80

ED indicates emergency department; IQR, interquartile range; MCR, missed case rate; STEMI, ST‐segment elevation myocardial infarction.

### Screening Performance: Missed Versus Captured Cases

**Figure 4:**
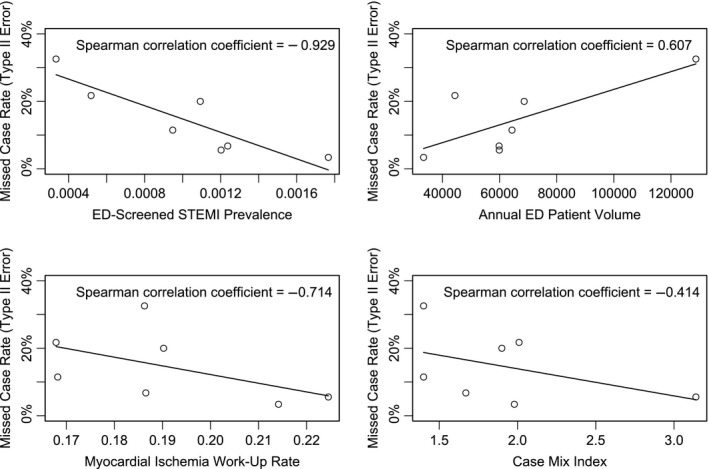
Unadjusted association between missed case rate and ED characteristics. ED indicates emergency department; STEMI, ST‐segment elevation myocardial infarction.

The overall MCR was 12.8% (95% CI 9.9–16.4%), representing the frequency of delayed or missed STEMI diagnoses (Table 1). We observed a range of 3.4% to 32.6% across the 7 EDs. The mean difference between the median door‐to‐ECG time for captured and missed primarily screened STEMI patients in each ED ranged from 14 to 80 minutes (Table 2). We found that the MCR was strongly associated with lower STEMI prevalence (*r*_s_=−0.929, range 0.04–0.13%, *P*=0.003). There was also a notable association with the myocardial ischemia work‐up rate (*r*_s_=−0.714, *P*=0.355). Higher annual patient volume (range 33 401–128 730) showed a moderately positive correlation (*r*_s_=0.607, *P*=0.148). Case mix index (range 1.4–3.14) demonstrated a moderate correlation (*r*_s_=−0.739, *P*=0.355) with the MCR. The comprehensiveness of the screening criteria (*r*_s_=0.30; range 3–5) for an early ECG and accuracy (*r*_s_=−0.214, *P*=0.645); range 81–92%) did not demonstrate significant correlations with the MCR or other screening performance characteristics (Figure 4).

### Quantifying Screening: Performance Variability

In addition to the variability noted in the MCR, the sensitivity and specificity of STEMI screening ranged from 65% to 97% and from 81% to 97%, respectively. Given the frequency with which ECGs are done and the rarity of STEMI in the broader ED population, the positive predictive value ranged from 0.29% to 5.06%, and the negative predictive value was nearly 1 with a range of 99.97% to 99.99%. Typically, this would be evidence of an excellent test;^27^ however, given the gravity of STEMI and the resources invested to avoid missing any cases, a measure that differentiates performance beyond these parameters is required. As noted earlier, screening accuracy ranged from 81% to 97%, but the diagnostic odds ratio was more discriminatory, with a range of 17 to 860. We found that the EDs with the greatest informedness (sensitivity+specificity−1) demonstrated superior performance across all of these screening measures.

### Comprehensiveness of Screening Criteria

Among the screening criteria, chest pain and atypical symptoms were used by all 7 EDs. Associated symptoms were included by 71%, and 86% included the consideration of an age threshold. Two EDs (ED 3 and ED 5) included all 4 screening criteria categories (Table 1) and had MCRs of 20% and 6.8%, respectively. These same EDs included other criteria in their screening process. ED 3 included considerations for the recent use of cocaine, history of a congenital heart defect, and coronary stent placement or cardiac surgery within 9 months. ED 5 included all patients on dialysis. Only ED 2 included a consideration of patient sex.

### Screening Process

Physicians were not involved in STEMI screening at any of the sites. A registered nurse was most frequently the person to both assess an arriving patient and document the patient's chief complaint. No sites used check‐in kiosks. The use of non–clinically trained registration clerks as the point of first contact was not associated with poorer performance by any of the screening test measures (Table 1). EDs with a triage registered nurse as the first point of contact for EMS arrival patients had lower MCRs compared with EDs with other first points of contact (*P*=0.034).

## Discussion

Our primary study finding was an overall MCR of 12.8% for primarily screened ED STEMI patients. This rate represents the frequency of missed STEMI screening, demonstrated by a delay in door‐to‐ECG time. In addition, the lowest and highest performing EDs had MCRs of 32.6% and 3.4%, respectively. This 28.2% difference is clinically meaningful because it suggests patients are exposed to variable risk of diagnostic delay depending on which ED they enter for care. In addition, the cost of ineffective screening results in missed cases experiencing 14 to 80 minutes of myocardial ischemia time. This ED‐level analysis is analogous to assessing the performance of a radiology or laboratory test in a population, in which the performance of 1 test is assessed as a summary of how accurately it identifies a disease in individual patients. Studies of this kind are often performed at a single center; however, we included 7 sites to strengthen our analysis and exploration of STEMI screening performance variation. In this way, we evaluated 7 different tests, serving the same function, in 7 patient populations, totaling 472 166 ED encounters.

For an acutely life‐threatening disease like STEMI, for which a missed case burdens the patient with significant negative sequelae, the reliability of a negative screen is of high value. The negative predictive value can often inform the performance of this “rule out” function. For a rare condition like STEMI, however, the negative predictive value will be ≈1 (Table 1). An alternative is the diagnostic odds ratio, which is not influenced by incidence. It is a traditional comparative measure of screening test quality across populations but is less clinically intuitive for the average clinician than the MCR. The MCR represents the tendencies communicated by sensitivity, specificity, accuracy, and the diagnostic odds ratio with a single measure. Consequently, we propose that the MCR is a more clinically meaningful and easily interpreted performance measure for primarily screened ED patients. Its name and calculation better communicate the high stakes of a missed STEMI case while informing an understudied area of STEMI screening performance.

Informedness should also be considered a valuable performance metric. With a STEMI prevalence of 0.09% (range 0.03–0.18%), increasing the sensitivity of screening alone will produce far more physician workflow interruptions for the ECG interpretation of false‐positive screens than it will improve the number of STEMI cases detected. This approach challenges the quality of care for the majority of patients because of the continual interruption of the physician's attention to interpret early ECGs. Targeting improvements in informedness (sensitivity+specificity−1) as a performance measure places value on achieving a balance between sensitivity and specificity in a manner analogous to the ideal cut point (Youden's point) on a receiver operating characteristic curve.^23^ In our study, informedness demonstrated a negative correlation with the MCR. The 2 EDs with the highest informedness measures (85% and 97% [range 57–97%]) had the lowest MCRs (3.4% and 5.6% [range 3.4–32.6%]), highest sensitivity (94% and 97% [range 67–97%]), highest specificity (91% and 97% [range 81–97%]), and highest diagnostic odds ratios (168 and 860 [range 17–171]). This suggests that informedness has value as a measure of the balance in sensitivity and specificity of an ED's screening criteria and as a marker of better STEMI screening performance. MCR and informedness were not tracked previously by these EDs.

Screening criteria comprehensiveness did not have the expected influence on the MCR. The EDs with the greatest number of screening criteria—ED 3 (MCR 20.0%, 95% CI 12.5–30.4%) and ED 5 (MCR 6.8%, 95% CI 2.9–14.9%)—did not have the highest screening performance. In addition, the ED with the lowest MCR (ED 7: MCR 3.4%, 95% CI 0.9–11.5%), representing the best performance, did not have the most comprehensive screening criteria. Reporting chest pain or atypical ischemia symptoms (shortness of breath, neck or shoulder pain) uniformly triggered an early ECG in all EDs. The lowest and highest performing EDs (ED 1 and ED 7, respectively) had equivalent numbers of categories included in their screening criteria (3). Their criteria differed in that the best performer included associated symptoms and the lowest performer included an age threshold. These results are contrary to our expectation based on the results of a 2012 study that used classification and regression tree analysis to identify ideal universal screening criteria to achieve the lowest MCR in an ED.^13^ Only 1 ED included a consideration of sex in its screening criteria despite the literature noting that male sex is a risk factor for STEMI and being female increases the likelihood of reporting non–chest pain symptoms.^8^, ^9^, ^10^ Our findings suggest that the screening environment influences performance. In addition, the incremental value of each criterion is not well understood and requires further investigation. Detecting a relationship will require a larger ED sample or a multicenter study using patient‐level data that includes screening criteria compliance, patient population, and institutional characteristics as confounders.

We anticipated that EDs with higher MCRs would have higher myocardial ischemia work‐up rates and that this would be associated with a higher case mix index and annual ED patient volume. We found the myocardial ischemia evaluation rate to have a strong association with the MCR; however, this finding was not statistically significant (*P*=0.07). Given our small sample size, this association should be explored in a larger study. Other associations with the MCR should be considered for further exploration. Case mix index had a moderate negative correlation with the MCR. This suggests the potential influence of patient population acuity. Annual ED volume had a moderately strong inverse relationship (*r*_s_=0.607, *P*=0.167) with the MCR. Higher volume may reflect larger centers with more experience identifying atypical presentations of STEMI; however, this is difficult to assess through this study because all study sites were academic tertiary care facilities. It may be the case that smaller EDs, which often have less robust triage processes or shorter wait times to see a physician, have lower MCRs. Conversely, EDs with lower annual patient volumes may see fewer cases of this rare diagnosis and have higher MCRs. This particular question warrants further study.

We observed less variation than we anticipated regarding (1) the point of first contact for primarily screened ED patients arriving via EMS (ie, ambulance) or self‐transport and (2) the ED staff documenting the reason for the ED visit during intake. A triage registered nurse was the most common staff member performing intake. None of our sites used a physician in triage as the first contact for patient intake. We do not believe the involvement of physicians in intake screening for STEMI is the solution to performance improvement. STEMI is a rare disease, and in EDs where many more patients require direct physician attention for more common conditions, such an approach could cause more harm than benefit. The demonstrated lack of physician clinical decision making in this arrival process and the more common use of non–clinically trained clerks emphasizes the importance of well‐developed diagnostic algorithms for timely screening and diagnosis.

Our study approach challenges the traditional characterization of ECG as a screening test for STEMI with cardiac catheterization angiography as the standard criterion. We contend that the ED screening criteria for an early ECG is the screening test. Despite its limited specificity for occlusive coronary thrombus,^25^ the early ECG is a diagnostic test used to determine whether or not to activate the catheterization lab emergently. Our findings highlight the need to investigate the diversity of screening criteria used in clinical practice with more scrutiny. This is particularly important given that the Guidelines Applied in Practice—Door to Balloon (GAP‐D2B) National Quality Initiative recommends ED physician activation of the catheterization lab for STEMI intervention as a priority process improvement.^28^, ^29^, ^30^ A multicenter patient‐level study is essential to better understand what criteria are needed for an individual ED to reduce its local MCR. This further investigation should be performed with broader diversity of ED types (community practice, rural, small and medium patient volume, non–trauma centers) using patient visit data (arrival time, early ECG time, STEMI diagnosis time, type of treatment delivered, time to treatment, clinical outcomes) that include both ED characteristics (ED volume, urbanicity, academic status, case mix index, percutaneous coronary intervention center status) and patient characteristics (age, sex, race, coronary artery disease risk factors, prior use of risk factor–modifying medications or intervention, literacy, numeracy, insurance status, socioeconomic status).

In addition, considering improvements in screening quality requires a patient‐centered perspective. The variable screening performance observed is not transparent to patients, referring providers, or prehospital personnel. This is primarily because existing STEMI registries and performance measures do not currently consider the quality of STEMI screening, nor do they account for the difference between the primarily screened ED STEMI patient and those who have been prescreened or prediagnosed prior to ED arrival.^11^, ^25^, ^31^ This distinction is necessary to inform the quality of case detection and to differentiate patients for whom the burden of ED evaluation includes the timely consideration of myocardial ischemia. In addition, quantifying screening performance in each of these patient populations is important because the burden of action for the receiving ED is different for each STEMI patient type. Many EDs have the routine practice of “rediagnosing” prediagnosed patients so the receiving local provider can ensure that treatment is initiated appropriately. Others will direct prediagnosed patients to the coronary catheterization lab without undergoing a repeat evaluation in the receiving ED. For time‐sensitive conditions like STEMI, stronger consideration should be given as to whether rediagnosis is needed with the added time delay. Future patient‐level studies should explore this question with considerations for the diversity of ED types and resource settings and for those receiving percutaneous coronary intervention versus thrombolysis.

### Limitations

Despite capturing STEMI screening for nearly half a million patients in a geographically diverse sample, our study includes only 407 patients with STEMI. This limits our ability to quantify the strengths of associations among ED characteristics, process, and screening performance; however, it is multicenter study focused on a subset of the larger hospital STEMI population with unique screening and diagnostic needs. We did not focus on a traditional clinical outcome. Rather, we identified novel performance measures for STEMI screening—the MCR and informedness—while considering univariate associations with contextual confounders. Each study ED uses an EMR system. This permitted reliable capture of clinical care events, time of ECG completion, and intervention. The structured event time stamping and uniform data extraction within these systems enabled quality retrospective data collection and case review. In addition, our selection of a prospective historical design reduced the potential influence of the Hawthorne effect in this process‐oriented study.

One must be cautious in comparing our reported MCR and STEMI prevalence with other STEMI performance studies, given our patient‐centered definition of a missed case and the limitation of STEMI screening to a subset of the larger STEMI patient population. In this study of patients primarily screened by the ED for STEMI, we sought to characterize how structured screening was associated with the timeliness of STEMI identification using aggregate case counts from each ED. A follow‐up study with patient visit–level data is needed to measure how consistently the local screening criteria were applied. Quantifying compliance in this manner was outside the scope of this study and cannot be informed by our study design. A patient‐level study will also enable the identification of differences among the prescreened, prediagnosed, and primarily screened patient populations and risk factors associated with untimely screening.

Last, we were able to collect characterizing data for each study site but did not fully characterize the EMS and referring environment. These factors likely influenced the MCR because in a surrounding community with robust prehospital care, a patient with more typical STEMI symptoms (chest pain) may be more likely to arrive prescreened or prediagnosed. This would have led to exclusion from our study population. This aspect is important to understand because the criteria used by EDs with more robust prehospital screening may need to be tailored to include more atypical presentations. A follow‐up study of the distribution of STEMI symptoms in the prescreened, prediagnosed, and primarily screened populations will help us understand the influence of prehospital care on screening performance.

## Conclusion

An overall MCR of 12.8% represents missed or delayed STEMI screening, as shown by delayed initiation of the diagnostic test, the ECG. Our observed MCR range difference of 29.2% demonstrates significant variability in the timely diagnosis of STEMI in primarily screened ED patients. The impact of ineffective screening is 14 to 80 minutes of myocardial ischemia time. Inferences drawn from the diagnostic odds ratio and the informedness of the individual EDs were generally consistent with comparisons based on the MCR alone. The ability of informedness to characterize a balance between improved case detection and false negatives makes it a measure to consider for tracking STEMI screening performance across EDs, along with the MCR. This exploration identified variation requiring further research and quality‐improvement attention that can be validated in a larger sample of EDs and patients in the future.

## Author Contributions

Dr Yiadom conceptualized and designed the study, and served as the lead primary investigator. She also coordinated data collection, analysis and interpretation. Conor McWade developed the online study data collection tool in REDCap, assisted with study coordination, and drafted the introduction and methods section of the manuscript. Drs Baugh, Tanski, Mills, Patterson, Salazar and Song served as study site PIs by organizing data collection from their institution's electronic medical record system and STEMI Case Review Committee, performing case adjudication for any discrepancies, and submitting study data to Dr Yiadom at the data coordinating center (Vanderbilt University). Cathy Jenkins was the biostatistician that assured consistency between the study design plan, study protocol, and data collection tool before study initiation. Dr X. Liu was the primary biostatistician for the study analysis. Dr D. Liu was the senior biostatistician for the study analysis and data presentation. Drs Wang, Dittus and Storrow contributed to the discussion, analysis, interpretation, and review of the manuscript. All co‐authors have reviewed, edited and approved the final draft of this manuscript.

## Sources of Funding

Dr Yiadom is Director of the ED Operations Study Group and supported by the National Heart, Lung, and Blood Institute's (NHLBI) Emergency Care K12 Research Training Program at Vanderbilt University. Research reported in this article was supported by National Heart Lung and Blood Institute of the National Institutes of Health, award number: 5K12HL109019, and the National Center for Advancing Translational Sciences/NIH UL1 TR000445. Dr Storrow is supported by NHLBI K12HL109019, NHLBI RO1HL111033, National Center for Advancing Translational Sciences/NIH UL1 TR000445, and PCORI FC14‐1409‐21656.

## Disclosures

Dr Storrow has received grant support from Abbott Diagnostics and Roche Diagnostics. He is a consultant for Roche Diagnostics, Novartis Pharmaceuticals Corp, Alere Diagnostics, Trevena, Beckman Coulter, and Siemens. The content is solely the responsibility of the authors and does not necessarily represent the official views of the National Institutes of Health.

## Supporting information


**Data S1.** Study protocol (amended).
**Table S1.** Early ECG Trigger Criteria Screening Performance
**Table S2.** 2×2 Contingency Table Data (Question Numbers Included)
