# Supplementary material for: Performance of Emergency Department Screening Criteria for an Early ECG to Identify ST‐Segment Elevation Myocardial Infarction
Source: J Am Heart Assoc. 2017 Feb 23;6(3):e003528. doi: 10.1161/JAHA.116.003528 (PMC5523988; doi:10.1161/JAHA.116.003528)
Supplement: Supplementary file 1 — Data S1. Study protocol (amended). Table S1. Early ECG Trigger Criteria Screening Performance Table S2. 2×2 Contingency Table Data (Question Numbers Included) [file JAH3-6-e003528-s001.pdf]

SUPPLEMENTAL MATERIAL

Data S1 – Study Protocol (AMMENDED)

Performance of Emergency Department Screening Trigger Criteria for an Early ECG to Identify STEMI

Primary Investigator: Maame Yaa A. B. Yiadom MD MPH, Emergency Medicine, Vanderbilt University
Data Coordinating Center: Vanderbilt University
Vanderbilt Key Study Personnel: Conor McWade MPH, Christina Kampe, Amy Diatikar, Pam Xu

Collaborating Site-PIs:	Chris Baugh MD MBA	Brigham and Women's Hospital
	Angela Mills MD	University of Pennsylvania
	Gilberto Salazar MD	University of Texas Southwestern
	Brian Patterson MD	University of Wisconsin
	Mary Tanski MD	Oregon Health & Sciences University
	KJ Song MD	University of California at Davis

INTRODUCTION

Background

ST-segment elevation myocardial infarction (STEMI) is a life threatening, time-sensitive condition consisting of a sudden complete blockage of a coronary artery.[1] It requires treatment within 30-90 minutes, and the diagnosis is made via electrocardiogram (ECG). Patients suspected of STEMI are referred to an emergency department (ED) for rapid evaluation, diagnosis, and treatment. Guidelines recommend the diagnosis be made within 10 minutes of arrival.[2] However, the first 10 minutes of an ED visit typically consist of registration and triage. The median door-to-doctor time for an ED patient is 60 minutes. As a result, ED registration and triage staff use screening criteria to identify patients who should receive an early ECG to identify STEMI. To date, the test characteristics of the screening process have not been extensively investigated. This study will examine whether there is variation in STEMI screening performance in different EDs, and whether variability is associated with the comprehensiveness of the screening criteria used.

Chest pain is the classic symptom for STEMI, however 20-30% of STEMI patients do not report chest pain.[3,4,5,6,7] Appropriately comprehensive STEMI screening criteria, used to trigger an early ECG (trigger criteria), should include more information than chest pain presence or absence. Ideal STEMI case capture occurs when trigger criteria also includes atypical symptoms like shortness of breath and dizziness; associated symptoms like jaw pain, neck pain or back pain; and considerations of the patient's age.[8,9,10] Previously collected data suggest the inclusion of these characteristics in different EDs trigger criteria varies significantly. We hypothesize there is variability in the screening processes between institutions, and this variability is associated with different diagnostic test characteristics.

Rationale

The ECG is often referred to as the screening test for STEMI. This is because 30-50% of patients with STEMI on ECG will not have an occlusive coronary artery thrombus when evaluated with coronary artery angiography, the criterion (gold) standard diagnostic test. Despite this limitation, the risk-benefit balance of early treatment verses further testing weighs in favor of early treatment. As a result, treatment is initiated based on the *reference diagnostic testing standard,* an ECG demonstrating STEMI. In the context of ED operations and patient flow, the trigger criteria is the

true screening test. Patients, who do not receive an early ECG from registration or triage, go on to receive typical ED care. This provides an opportunity to identify cases missed by the index test, making typical ED care an *alternative reference standard* for trigger negative patients.[10,11]

We have looked at the STEMI screening performance at Vanderbilt University (Table S1). In doing so we considered a patient meeting trigger criteria, which prompts the ED registration or triage staff to initiate an early ECG, as a positive *index test*; and an ECG indicating STEMI as a positive *reference test*. We found that the screening process has high sensitivity and negative predictive value with poor positive predictive value and limited specificity. The likelihood ratio of positive test is 5.2 (probability of a person with a STEMI ECG screening positive via screening trigger criteria), and that of a negative test is 0.025 (probability of a person with a STEMI ECG not being identified with screening trigger criteria). This study seeks to understand these local results in the context of broader clinical practice and variability of trigger criteria between institutions.

Study Objectives

This study has two objectives. It will first examine whether the STEMI screening performance observed at Vanderbilt is generalizable. We will do this by calculating 2x2 contingency table data for patients seen in six additional EDs (in different regions of the country) individually, and then as a total population combined with Vanderbilt. Second, it will determine whether STEMI screening performance improves with more data incorporated into the trigger criteria by identifying whether there is an association between positive and negative likelihood ratios with the inclusion of associated symptoms, atypical symptoms, and age in the trigger criteria.

Potential Risks and Benefits

The ED is the unit of observation in this study. As a result, the data consists of aggregate numbers representing ED patient care events in 2014. Protected health information (PHI) and patient level data may be reviewed within the local ED to get the aggregate numbers we will use to calculate screening performance. Related data extraction will use the administrative reporting functions within the hospitals' electronic medical record systems (EMR) and review of existing summative data reports from the hospitals' STEMI case review committees. As a result there is limited potential for study-related breaches in PHI confidentiality, thus presenting minimal risk to the EDs and their patients. The participating EDs are involved in this study as a quality improvement effort with the intent to publish the results as research. The benefit to the EDs, and the patients they serve, is gaining knowledge about their local practice in the context of national peers to inform their current clinical STEMI screening practices. There is no compensation for participation.

OBJECTIVES AND OUTCOME MEASURES

This study will see whether the Vanderbilt results are generalizable by calculating 2x2 table data (see Table 1) for 7 EDs in different regions of the United States. Specifically, we will identify the sensitivity, specificity, negative predictive value, positive predictive value, false negative rate, false positive rate, likelihood ratio of being screened for an early ECG if the patient's ECG shows STEMI, likelihood ratio of triggering an early ECG if the patient's ECG is negative.

STUDY DESIGN

Design

Cross sectional, retrospective cohort, multi-centered study

Subject Inclusion

All patients screened for an early ECG to identify STEMI in 2014 in seven different EDs

Data Collection

A site-PI from each ED will be responsible for extracting aggregate figures included in the study survey (Appendix I) using data from their EMR reporting functions and their hospital STEMI case review committee. To reduce misclassification bias, STEMI is defined using ICD-9 codes previously validated in the literature (410.01, 410.11, 410.21, 410.31, 410.41, 410.51, 410.61, 410.81, 410.91).[12] The EMR will provide aggregated numbers on ED care events (number of patients seen, number of patients receiving an early ECG as a result of triggering the screening criteria, etc.), while the hospital STEMI committee report will provide verifying counts for all ED STEMI cases, whether the early ECG demonstrated STEMI, and missed ED STEMI cases. For any discrepancies between the EMR and STEMI committee reports, the site PI will review the associated patient charts and make a clinical decision on whether the case was Trigger positive or negative, and STEMI positive or negative. The survey is included as appendix I, and will be sent to the site-PIs via email using REDCap. REDCap will also serve as a secure study data repository. The questionnaire has been reviewed by a biostatistician for completeness and to ensure it appropriately supports the planned analysis. (see attached)

Study Timeline

The study will begin upon receipt of IRB approval from all participating sites. We anticipate data collection will take four weeks. Analysis will begin upon receipt of data from all participating sites. The data will be analyzed with the assistance of the Department of Biostatistics at Vanderbilt.

Enrollment

Sites will be enrolled upon the submission of a completed survey and institutional IRB approval letter.

Confidentiality and Source Data

Only key study personnel at the Data Coordinating Center, Vanderbilt University, will have access to the study data from all 7 EDs.

SAMPLE SIZE

Seven EDs will be included. In 2014 these EDs saw 64353, 59929, 53165, 155000, 68621, 46378, 60000 patients. This results in a total of 507446 patients screened for STEMI. Based on previously reported data, and Vanderbilt's 2014 data, the incidence of STEMI is approximately 0.14-0.18%.[8,12] Using the median estimate of 0.16% we anticipate 812 STEMI cases.

FINAL ANALYSIS PLAN

Two-by-two contingency tables will be calculated for each ED, and all EDs combined (Table S2). The data will be reported as sensitivity, specificity, negative predictive value, positive predictive value, false negative rate, false positive rate, likelihood ratio of being screened for an early ECG (positive index test) if the ECG demonstrates STEMI (positive reference test), likelihood ratio of being screened for an early ECG (positive index test) if the ECG does not show STEMI (negative reference test). This data will then be correlated with the inclusion of atypical symptoms, associated symptoms or age (representing yes or no as binary values) in the trigger criteria via a multi-variate logistic regression to see if there is an association.

STUDY OUTCOME

- Estimates for the screening performance of ED trigger criterion for an early ECG to identify STEMI including sensitivity, specificity, NPV, PPV, LR+, LR-, and the missed case rate (false negative rate)
- Description of whether the comprehensiveness of the criteria influences screening performance.

SIGNIFICANCE

This will be the first study to evaluate ED STEMI trigger criteria screening performance across institutions in different regions of the United States.

PERFORMANCE OF STEMI SCREENING TRIGGER CRITERIA

Online form available at:https://redcap.vanderbilt.edu/surveys/?s=ET3WK99HDM

The electrocardiogram (ECG) is often described as a screening test for ST-elevation myocardial infarction (STEMI), with coronary angiography as the criterion standard diagnostic test. However, in the emergency department (ED), treatment is initiated based on the presence of STEMI on ECG. In addition, timely diagnosis should be made within 10 minutes, well before a patient is seen by a physician and during the processes of registration and triage. As a result, in the context of ED patient care processes and flow, the ECG is the ***diagnostic test*** and the clinical criteria used to screen arriving ED patients for STEMI in during registration and triage (ECG trigger criteria) is the true ***screening test***.

This study seeks to quantify the screening performance of the early ECG trigger criteria used to screen for STEMI across 7 geographically diverse EDs. The screening population includes all ED patients ≥ 18 years of age seen in the ED from January 1, 2014 at 12:00am to December 31, 2014 at 11:59am. It assumes that all ECGs done within the first 15 minutes of an ED visit are to screen for STEMI. The ***STEMI screening criteria*** is the index test. Triggering an early ECG is a positive index test. An ***early ECG*** (performed ≤15 minutes after patient arrival) is the reference diagnostic test standard. An ECG demonstrating STEMI is a positive reference test. Patients, who do not receive an early ECG from registration or triage, go on to receive typical ED care. This provides an opportunity to identify cases missed by the index test, making typical ED care an alternative reference standard for trigger negative patients.[3,14]

Key Definitions

Trigger positive = early ECG to screen for STEMI = an ECG performed ≤ 15 minutes of arrival
Arrival = first documented presence or time-stamp of the patients presence in the ED
EMS = patients coming to the ED via emergency medical system (EMS) ambulance or helicopter
Walk-in = patients coming to the ED via non-EMS transportation

Data Collection Sources

ED and hospital electronic medical record, or summative case counts from a hospital STEMI Case Review Committee

---

SURVEY

1) ED Name: [drop down menu]
- Select one
- Brigham and Women’s Hospital
- Hospital of the University of Pennsylvania
- Vanderbilt University
- University of Wisconsin
- University of Texas Southwestern
- Oregon Health & Sciences University
- University of California - Davis

2) Please upload a copy of your IRB approval letter as a pdf file.

3) In 2014, how did you identify arriving ED patients for an early ECG to screen for STEMI?

[drop down menu]
- Select one
- Established registration/triage criteria or protocol
- Solely at the discretion of the registration staff or triaging provider

**EDOSG Baseline Data Collection Tool Question #41*

4) If you have an established early ECG protocol that serve as your "trigger criteria," upload a PDF or MS Word document including the criteria/policy for which patients get an early ECG to screen for STEMI.

5) In addition to "chest pain" what elements are included in your early ECG screening trigger criteria to identify patients for an early ECG? Select all that apply:
○ Atypical STEMI symptoms (shortness of breath, dyspnea on exertion, fatigue, nausea)
○ Associated STEMI symptoms (arm pain, jaw pain, back pain, epigastric pain, abdominal pain)
○ An age threshold
○ Other
○ Not applicable. We do not have an established trigger criteria

6) What was the total number of patients, ≥18 years of age, seen in the ED in 2014? Include all individuals registered as ED patients. Do not exclude the following ED dispositions: eloped, left without being seen, left before completing treatment, left AMA.
<<Free text response>>

7) In 2014, what was the total number of patients (≥18 years of age) that had an ECG performed ≤15 minutes after their arrival in the ED?
<<Free text response>>

8) What was the total number of 2014 STEMI cases that came through the ED? Include all patients, ≥18 years of age, who were registered as ED patients? This is either all ED patients with an ED admission or hospital discharge diagnosis of STEMI (via an electronic medical record query for diagnosis "STEMI" or ICD9 codes 410.01, 410.11, 410.21, 410.31, 410.41, 410.51, 410.61, 410.81, 410.91).[13] Confirm this number with your hospital STEMI Case Review Committee. If there is a discrepancy, review the individual patient charts. Based on Vanderbilt data, the incidence of STEMI reported in the literature, and all participant ED patient volumes, there should be no more than 5 cases that require review.
<<Free text response>>

9) What was the total number of 2014 STEMI cases that came through the ED that <u>did not</u> get an early ECG (ECG ≤ 15 minutes of arrival)? These are the missed screening cases. This can be obtained as an adjudicated case count from your hospital STEMI committee. Many STEMI quality improvement committees will document time-to-ECG permitting an assessment of those missed by screening. These should not include patients whose were screened, but had STEMI-negative early ECGs.
<<Free text response>>

10) What was the total number of 2014 ED patients who had both an ECG performed and a serum troponin ordered and resulted. This is an estimate of the underlying "chest pain patient" burden.
<<Free text response>>

	Greeter	Check-in Kiosk	Registration Clerk	Triage RN	Midlevel Provider	Physician	Other
11) Who/what is the first contact for ED patient brought in by EMS? (select one)	○	○	○	○	○	○	○
12) Who/what records EMS arrival patients' presenting chief complaint? (select one)	○	○	○	○	○	○	○
13) Who/what is the first contact for arriving ED patients what walk-in? (select one)	○	○	○	○	○	○	○
14) Who/what records walk-in patients' presenting chief complaint? (select one)	○	○	○	○	○	○	○

15) How many hours did it take to collect the data? Please include all time spent meeting, generating reports, extracting and cleaning data by you or your proxy (IT staff or assistant). Exclude time preparing your IRB application. Round up to the nearest hour.

16) What is the median Door- to-ECG time for all primarily screened ED patients with a final hospital diagnosis of STEMI?

17) What is the 25th percentile Door-to-ECG time for all primarily screened ED patients with a final hospital diagnosis of STEMI?

18) What is the 75th percentile Door-to-ECG time for all primarily screened ED patients with a final hospital diagnosis of STEMI?

19) What is the median Door- to-ECG time for all primarily screened ED patients with a final hospital diagnosis of STEMI, who received their ECG ≤15 minutes of ED arrival?

20) What is the 25th percentile Door-to-ECG time for all primarily screened ED patients with a final hospital diagnosis of STEMI, who received their ECG ≤15 minutes of ED arrival?

21) What is the 75th percentile Door-to-ECG time for all primarily screened ED patients with a final hospital diagnosis of STEMI, who received their ECG ≤15 minutes of ED arrival?

22) What is the median Door- to-ECG time for all primarily screened ED patients with a final hospital diagnosis of STEMI, who received their ECG >15 minutes of ED arrival?

23) What is the 25th percentile Door-to-ECG time for all primarily screened ED patients with a final hospital diagnosis of STEMI, who received their ECG >15 minutes of ED arrival?

24) What is the 75th percentile Door-to-ECG time for all primarily screened ED patients with a final hospital diagnosis of STEMI, who received their ECG >15 minutes of ED arrival?

25) What was your hospital Case Mix Index (CMI) for 2014?

Table S1: Early ECG Trigger Criteria Screening Performance

Vanderbilt University Medical Center		***Truth***		
		STEMI		
		(+)<br>null false	(-)<br>null true	
Test (Screening Criteria)	Screen (+)	**60**<br>true positive	**13,092**<br>false positive (Type I Error)	13,152
	Screen (-)	**15**<br>false negative (Type II Error)	**55,454**<br>true negative	55,469
		75	68,546	68,621
		Sensitivity (1-β)<br>80%	Specificity<br>81%	Accuracy<br>81%

Table S2: 2x2 Contingency Table Data (Question numbers included below)

		Disease<br>ECG = Reference Test		
		**STEMI (+)**	**STEMI (-)**	
Test<br>Trigger Criteria<br>= Index Test	**Trigger (+)**	#8-#9	#7-#8+9	#7
	**Trigger (-)**	#9	#6-#7-#9	#6-#7
		#8	#6-#8	#6

PROTOCOL REFERENCES:

1. Yiadom, MYAB. Emergency department treatment of acute coronary syndromes. *Emergency Medicine Clinics of North America.* 2011;4: 699-710.

2. O'Gara PT, Kushner FG, Ascheim DD, Casey DE, Chung MK, de Lemos JA, Ettinger SM, Fang JC, Fesmire FM, Franklin BA, Granger CB. 2013 ACCF/AHA guideline for the management of ST-elevation myocardial infarction: executive summary: a report of the American College of Cardiology Foundation/American Heart Association Task Force on Practice Guidelines. *JACC.* 2013;4: e78-e140

3. Canto JG, Shlipak MG, Rogers WJ, Malmgren JA, Frederick PD, Lambrew CT, Ornato JP, Barron HV, Kiefe CI. Prevalence, clinical characteristics, and mortality among patients with myocardial infarction presenting without chest pain. *JAMA.* 2000;283:3223-3229.

4. Blomkalns AL, Chen AY, Hochman JS, Peterson ED, Trynosky K, Diercks DB, Brogan GX, Boden WE, Roe MT, Ohman EM, Gibler WB. Gender disparities in the diagnosis and treatment of non–ST-segment elevation acute coronary syndromes: large-scale observations from the CRUSADE (Can Rapid Risk Stratification of Unstable Angina Patients Suppress Adverse Outcomes With Early Implementation of the American College of Cardiology/American Heart Association Guidelines) national quality improvement initiative. *JACC.* 2005;45:832-7.

5. Milner KA, Vaccarino V, Arnold AL, Funk M, Goldberg RJ. Gender and age differences in chief complaints of acute myocardial infarction (Worcester Heart Attack Study). *Am J Cardiol.* 2004;93:606-608.

6. Dey S, Flather MD, Devlin G, Brieger D, Gurfinkel EP, Steg PG, FitzGerald G, Jackson EA, Eagle KA. Sex-related differences in the presentation, treatment and outcomes among patients with acute coronary syndromes: the Global Registry of Acute Coronary Events. *Heart.* 2009;95:20-26.

7. Brieger D, Eagle KA, Goodman SG, Steg PG, Budaj A, White K, Montalescot G. Acute coronary syndromes without chest pain, an underdiagnosed and undertreated high-risk group: insights from the Global Registry of Acute Coronary Events. *Chest.* 2004;126:461-469.

8. Panju AA., Hemmelgarn BR, Guyatt GH, Simel DL. *JAMA.*1998;280:1256-1263.

9. Glickman SW, Shofer FS, Wu MC, Scholer MJ, Ndubuizu A, Peterson ED, Granger CB, Cairns CB, Glickman LT. Development and validation of a prioritization rule for obtaining an immediate 12-lead electrocardiogram in the emergency department to identify ST-elevation myocardial infarction. *Am Heart J.* 2012;163:372-382

10. Lijmer JG, Mol BW, Heisterkamp S, Bonsel GJ, Prins MH, van der Meulen JH, Bossuyt PM. Empirical evidence of designs –related bias in studies of diagnostic tests. *JAMA.* 1999;282:1061-1066.

11. Carrington RM, Lachs MS, Feinstein AR. Use of Methodological standards in diagnostic test research: Getting better but still not good." *JAMA.*1995;274: 645-651.

12. Ward MJ, Kripalani S, Zhu Y, Storrow AB, Dittus RS, Harrell FE and Self WH. Incidence of emergency department visits for ST-elevation myocardial infarction in a recent six-year period in the United States. *Am J Cardiol.* 2015;115: 167-170.

13. Carrington RM, Lachs MS, Feinstein AR. Use of Methodological standards in diagnostic test research: Getting better but still not good." *JAMA.*1995;274: 645-651.
